# Early exposure hypothesis: where are we now?

**DOI:** 10.1186/2045-7022-1-S1-S71

**Published:** 2011-08-12

**Authors:** Gideon Lack

**Affiliations:** 1 St Thomas’ Hospital, Children's Allergies Department, Paediatric Allergy, King's College London; 2 Allergy Service, Guy’s & St Thomas’ NHS Foundation Trust, London, UK

## 

Despite increasing efforts to prevent food allergies in children, IgE-mediated food allergies continue to rise in westernized countries. The World Health Organisation recommends that during the first six months of life, infants be exclusively breast fed, and that weaning onto solids and milk formula should only occur after that period. Specific avoidance of foods such as egg and peanut in atopic infants has been believed to prevent the development of these allergies. While there is data suggesting that exclusive breast-feeding and the use of extensively hydrolyzed formula may prevent the development of eczema, there is insufficient evidence that such dietary interventions prevent the development of IgE-mediated food allergy. The dual exposure to allergen hypothesis posits that tolerance to antigens occurs in the neonate through high-dose oral exposure and that allergic sensitisation occurs through low dose cutaneous exposure. It has been shown that exposure of mice to ovalbumin or peanut on abraded skin led to significant specific IgE responses. In human subjects, there are studies in which food allergen–specific T cells have been isolated from lesional skin in patients with eczema. In a prospective birth cohort study it was found that low-dose exposure to peanut in the form of arachis oil applied to inflamed skin on infants was associated with increased risk of peanut allergy at age 5 years. Recent studies suggest that infants who are exposed to food allergens early through the oral route are less likely to have food allergies than infants without such exposure, but such observational cohort studies are subject to confounding and the possibility of reverse causality. An ongoing randomized trial involving infants at high risk for food allergy is comparing early exposure to high doses of food allergens with complete avoidance of these allergens during infancy (http://www.leapstudy.co.uk). The concept of oral tolerance induction to food allergy is being further investigated in another randomized controlled study to determine whether early weaning and exposure to food allergens (from 3 months of age) prevents the development of food allergies (http://www.eatstudy.co.uk).

